# True Progress, Moral Training in Medical Schools

**Published:** 1883-12-20

**Authors:** T. S. Powell


### *TRUE PROGRESS, MORAL TRAINING IN MEDICAL SCHOOLS.*

The dawn of 1884 will shed its light upon the grave of a dead year, that has been pregnant with many successful achievments in art, science, literature and general material prosperity. The ever onward movement of the revolving years, is typical of the incessent activity in all animate nature. But while seasons come and go—the vernal freshness of spring, the roses of summer, the "harvest home" under autumn skies, and the chilling blasts of the snow-shrouded winter man alone is dissatisfied with his condition, and fixes his eye on some progressive step that will insure successful results to the desire of his heart's ambition.

We fear though that among the majority of people the word "progress" is not truly understood. The idea seems too much to prevail that to progress, simply means to go ahead—to push on—dash every obstacle aside, and reach the desired goal of ambition, whether by legitimate means or not, so the object in view is attained at last.

But this is a very erroneous conception of successful achievments in any advanced movement that will benefit and elevate individuals or mankind at large.

True progress consists—first, in a worthy object to attain ; next, a plan of comprehensive, practical and honorable means by which to prosecute the work ; and, lastly, an energetic spirit that will never dispond, but grasp every lawful opportunity with a hopeful, undaunted enthusiasm, and use every legitimate facility that will aid in reaching the very highest possible attainment of the object in view, which object should never be for selfish purposes and personal aggrandizement, but for the welfare of humanity in the aggregate. In a word, progress, ostensibly for the broader civilization of the human race, is an evil rather than a good, unless its chief motive-power is a strong moral force. Every intelligent observer of men, morals and measures in this country, during the last fifteen or twenty years of our progressive civilization, will at once see the indisputable truth of this assertion.

Though much has been said and written upon a higher standard in medical education, we do not think the profession at large is fully aroused upon this important subject. We talk, and plan, and execute for progress in medicine, so as to push on and advance with the spirit of the age, but in doing so we are losing sight of many characteristics of the physicians of the "old school" which are indispensable to give an elevated tone to the standard of medical education, and to keep unsullied the code of ethics instituted and nobly lived out by so many of our illustrious predecessors.

Among these characteristics are cordial and undeviating professional courtesy, dignity of principles, strictly honorable methods, and the sacred obligations of the fraternal bond of union of the medical profession.

Retrogression in these respects, no doubt, is due to defects in both home and school training—to the decline of private and public moral sentiment—to the absence of high principles in the methods and actions of men, and of that reverent spirit for truth, justice, honor, fair dealing, and all the noble attributes of humanity, without which no man can properly estimate the character of another man, though he be unspotted by moral corruption.

The celebrated Dr. Channing has said that no human being, man or woman, can act up to a sublime standard without giving offence; and it is also notable that more men, perhaps, are persecuted for doing right and adhering to the truth, than for doing wrong and propagating falsehood.

Facts like these show that the moral statue of mankind is lamentably deficient in proportions; but he who in his life-work endeavors to exalt this statue to a more lofty height is the true hero in the ranks of humanity—the man of noble action and sublime moral courage.

The preliminary education of the student of medicine should not be obtained only from books. It should be broad and liberal, and of such a nature as to enable him to comprehend with a charitable, but intelligent spirit, the methods and actions of men—to discriminate between the evil and good in nature, and to choose the right in all things; not only because of its honor, purity and refining influence, but because all true and lasting success is the doing well whatever you do, with the solid ground of truth and integrity for the enduring basis of the work.

The student of medicine will then be prepared to aspire to the highest attainments in medical science, and to labor for that end with an upright, conscientious motive, and will illustrate in the profession the high standard of our ethical code. The young profession thus trained and prepared will also be enabled to see that an honorable competition between schools of medicine, is right and proper, and that the emulation of a higher education in these schools should elicit commendation from the whole profession, rather than discourtesy, injustice and underhanded methods from any of its members; all of which stains the escutcheon of medical jurisprudence and lowers the tone and dignity of its character.

Colleges which, by misrepresentations, etc., entice students from other schools of medicine, or obtain patronage by any other dishonable methods, do so in violation of our code of ethics; and such action must, perforce, exert an evil influence over both students and faculties, and disseminate demoralization throughout the professional ranks. Such schools should not be encouraged. but condemned by our journals and by the public. No matter how high the position a physician may hold in medical circles, he should have no more license to violate the laws that govern the profession than the most humble practitioner.

The observance of our code should be equal and uniform throughout the professional world, and the public in general, as not less than the profession, ought better to understand our ethical law, and encourage such institutions of medical training as are upright and honorable in all their methods and measures.

While medical schools should be charitable towards each other, and maintain inviolate our articles of jurisprudence, yet they have the right to properly advertise their institutions and fully set forth their claims in our journals, in circulars and newspapers, or in any dignified manner. Believing thus, we have not hesitated to advertise the SOUTHERN MEDICAL COLLEGE, of Atlanta, Georgia, by all honorable methods.

The members of the Faculty have honestly endeavored to put its merits and its claims before the public, and as it has been, from the beginning, their purpose and ambition to emulate in this school the best methods of medical education, characterized by a pure moral tone, the profession ought, and we believe will, encourage and sustain an institution that strives to be worthy of a discriminating and liberal patronage. T. S. P.

